# The application of cytokeratin-18 as a biomarker for drug-induced liver injury

**DOI:** 10.1007/s00204-021-03121-0

**Published:** 2021-07-29

**Authors:** Samantha Korver, Joanne Bowen, Kara Pearson, Raymond J. Gonzalez, Neil French, Kevin Park, Rosalind Jenkins, Christopher Goldring

**Affiliations:** 1 Department of Pharmacology and Therapeutics, Institute of Systems, Molecular and Integrative Biology, MRC Centre for Drug Safety Science, University of Liverpool, Liverpool, UK; 2 Adelaide Medical School, Faculty of Health and Medical Sciences, The University of Adelaide, Adelaide, Australia; 3 Merck and Co. Inc, Kenilworth, NJ USA

**Keywords:** Drug-induced liver injury (DILI), Hepatotoxicity, Cytokeratin-18 (CK18), Biomarker, In vivo

## Abstract

Drug-induced liver injury (DILI) is a frequent and dangerous adverse effect faced during preclinical and clinical drug therapy. DILI is a leading cause of candidate drug attrition, withdrawal and in clinic, is the primary cause of acute liver failure. Traditional diagnostic markers for DILI include alanine aminotransferase (ALT), aspartate aminotransferase (AST) and alkaline phosphatase (ALP). Yet, these routinely used diagnostic markers have several noteworthy limitations, restricting their sensitivity, specificity and accuracy in diagnosing DILI. Consequently, new biomarkers for DILI need to be identified.

A potential biomarker for DILI is cytokeratin-18 (CK18), an intermediate filament protein highly abundant in hepatocytes and cholangiocytes. Extensively researched in a variety of clinical settings, both full length and cleaved forms of CK18 can diagnose early-stage DILI and provide insight into the mechanism of hepatocellular injury compared to traditionally used diagnostic markers. However, relatively little research has been conducted on CK18 in preclinical models of DILI. In particular, CK18 and its relationship with DILI is yet to be characterised in an in vivo rat model. Such characterization of CK18 and ccCK18 responses may enable their use as translational biomarkers for hepatotoxicity and facilitate management of clinical DILI risk in drug development. The aim of this review is to discuss the application of CK18 as a biomarker for DILI. Specifically, this review will highlight the properties of CK18, summarise clinical research that utilised CK18 to diagnose DILI and examine the current challenges preventing the characterisation of CK18 in an in vivo rat model of DILI.

## Introduction

### Drug-induced liver injury (DILI)

Drug-induced liver injury (DILI) was first described in the 1960’s and was once considered a ‘penalty for progress’ (Popper et al. 1965). Today, DILI is a frequent and serious safety issue faced by clinicians, pharmaceutical companies and regulatory bodies. The liver plays a key role in first-pass metabolism and drug elimination, so it is often exposed to high drug concentrations. These factors are believed to be the predominant reasons why the organ is particularly susceptible to drug-induced injury (Atienzar et al. 2016).

DILI defines an array of drug-induced hepatocellular injuries ranging from acute or chronic hepatitis to acute liver failure and is referred to as either intrinsic or idiosyncratic (Alempijevic et al. 2017; Mayoral et al. 1999; O’Grady et al. 1993). Intrinsic DILI is predictable and dose-dependent, with hepatocellular injury attributed to the pharmacological or toxic properties of the drug (Alempijevic et al. 2017; McGill and Jaeschke 2019). Drugs such as acetaminophen (APAP) demonstrate intrinsic DILI, with hepatocellular injury following APAP overdose a result of excessive accumulation of APAP reactive metabolite *N*-acetyl-p-benzoquinoneimine (NAPQI) (Alempijevic et al. 2017). In comparison, idiosyncratic DILI is more complex due to its non-dose-dependent and varied nature, commonly attributed to hypersensitivity reactions, metabolic mechanisms of injury and patient genetic variation (Alempijevic et al. 2017; Fisher et al. 2015; McGill and Jaeschke 2019). The preclinical and clinical diagnosis of DILI is reliant on traditionally used biomarkers.

### Traditional biomarkers for DILI

The diagnostic principles for DILI remain unchanged from the 1960’s, with traditional biomarkers alanine aminotransferase (ALT), aspartate aminotransferase (AST), alkaline phosphatase (ALP) and total bilirubin (TBIL) still routinely utilised to diagnose DILI (Alempijevic et al. 2017; Church and Watkins 2017; Robles-Diaz et al. 2014). These biomarkers form the foundation of ‘Hy’s Law’ in which drug-induced hepatocellular injury is defined as; the presence of a threefold or greater elevation above the upper normal limit (ULN) of ALT or AST compared to control, the elevation of TBIL > 2 times the ULN without initial evidence of cholestasis and, no pre-existing or underlying explanation for the elevation of ALT, AST and TBIL, such as viral hepatitis (US Food and Drug Administration 2009). ALP is also applied to identify cholestasis, although a significant ALP level indicative of cholestasis is not clearly defined (Watkins et al. 2008). Although heavily utilised, these traditional biomarkers for DILI have many preclinical and clinical limitations affecting their sensitivity, specificity and accuracy in diagnosing DILI.

### The preclinical and clinical limitations of traditional biomarkers for DILI leave significant gaps in knowledge

A major preclinical limitation of traditional biomarkers for DILI faced during drug development is that ALT, AST and ALP are not specific to hepatocellular injury. In nonclinical species, ALT levels > 3–5 times the ULN are indicative of adverse hepatocellular injury, even in the absence of histological changes (US Food and Drug Administration 2009). During clinical investigations, the US Food and Drug Administration (FDA) recommends the discontinuation of preclinical drug development when serum ALT or AST levels reach > 8 of the ULN during treatment (US Food and Drug Administration 2009). Additionally, if serum ALT or AST levels are > 5 of the ULN for more than a 2-week period, with the appearance of fatigue, nausea, vomiting, right upper quadrant pain or tenderness, fever and/or rash in a clinical setting, the FDA also recommends discontinuation of treatment (US Food and Drug Administration 2009). Although ALT, AST and ALP are predominately found in the liver, they are also found in the kidneys, heart, brain, skeletal muscle and red blood cells (Church and Watkins 2017; Tajima et al. 2019). As such, increases in these biomarkers may not be indicative of hepatocellular injury or DILI, but of other forms of toxicity, such as, rhabdomyolysis or myocardial damage (Church and Watkins 2017; Tajima et al. 2019). While increased levels of traditional biomarkers for DILI are coupled with physical symptoms as previously described, these physical symptoms may also be indicative of other toxicities. Additionally, it is known that individuals can experience transient, non-adverse, fluctuations in ALT, AST and ALP levels (Church and Watkins 2017; Tajima et al. 2019). Several drugs in preclinical development have been discontinued due to significant elevations in traditional serum biomarkers of hepatotoxicity with no clear pathophysiological evidence of hepatocellular injury (Church and Watkins 2017; Tajima et al. 2019).

On the other hand, traditional biomarkers for DILI have also failed to identify some hepatoxic drugs prior to FDA approval and subsequent release to market. The FDA have withdrawn several drugs, such as bromfenac (non-steroidal anti-inflammatory), ebrotidine (H2-receptor antagonist) and troglitazone (PPAR activator), from market due to severe patient morbidity and mortality as a direct result of DILI (Hunter et al. 1999; Kohlroser et al. 2000). Notably, troglitazone (brand name Rezulin^®^, once prescribed for treatment of type 2 diabetes) demonstrated ALT > 3 ULN in 1.9% of patients with no reports of acute liver failure or severe hepatocellular injury throughout clinical trials (Goldkind and Laine 2006; Kohlroser et al. 2000; Mayall and Banerjee 2014). Mere months after its release to market, the FDA recommended monthly liver function tests following numerous reports of hepatic failure and liver transplantation in patients taking troglitazone (Aronson 2016; Kohlroser et al. 2000). Liver biopsies confirmed histopathological damage, such as necrosis and fibrosis, which subsided following cessation of troglitazone (Aronson 2016; Kohlroser et al. 2000). With reported cases of hepatic failure and liver transplantation steadily increasing, the FDA withdrew Rezulin^®^ in 2000 due to the life threatening hepatoxicity associated with the drug (Aronson 2016; Goldkind and Laine 2006; Mayall and Banerjee 2014). A key challenge in the case of troglitazone was that although traditional diagnostic markers for DILI identified significant hepatocellular injury, they were unable to shed light on potential mechanisms driving the observed injury.

Traditional biomarkers for DILI also do not provide insight into the mechanism of hepatocellular injury, a limitation for both preclinical and clinical assessment of drugs. Increased ALT, AST and ALP enzymatic activity in circulation is a direct result of increased tissue breakdown, but this gives no indication of how that damage occurred (Church and Watkins 2017; Tajima et al. 2019). The biological mechanism of hepatocellular injury is dependent on the type of drug and includes, but is not limited to, mitochondrial toxicity, reactive metabolite generation and oxidation (Church and Watkins 2017; Tajima et al. 2019). Initial hepatocellular injury may also be exacerbated, with activation of the innate and adaptive immune responses leading to further hepatocellular damage, such as fibrosis and hepatitis (Church and Watkins 2017; Tajima et al. 2019). For the majority of hepatoxic drugs and especially during preclinical drug development, the biological mechanism of hepatocellular injury is relatively unknown or poorly understood. A key issue in the area of drug development is the ability to translate hepatotoxicity findings in preclinical species to the likely risk of DILI in humans. In the clinic, understanding the biological mechanism of hepatocellular injury could help the diagnosis and treatment of DILI, allowing targeted therapy to improve overall prognosis. For example, if the mechanism of hepatocellular injury is identified to be predominately of an inflammatory nature, a corticosteroid could be administered to reduce the likelihood of further hepatocellular damage. However, this approach relies on early diagnosis of DILI, which is challenging when relying on traditional biomarkers for DILI.

A major clinical limitation of traditional DILI biomarkers involves interpretation of Hy’s Law and the associated levels of ALT, AST and ALP upon which a patient is determined to have DILI. As previously discussed, due to the nature of ALT, AST, ALP and TBIL, Hy’s Law identifies patients at high risk of fatal DILI, which is approximately 10% of all DILI cases (Robles-Diaz et al. 2014; Tajima et al. 2019). Therefore, the majority of patients with early-stage DILI are not diagnosed in the clinic, and it is critical that patients with early-stage DILI are identified. This would not only enable rapid and effective intervention, but would also improve long-term prognosis. DILI due to acetaminophen (APAP) overdose is a well-recognised and a frequent example of this. APAP associated hepatotoxicity is considered dose-dependent therefore, when used at therapeutic doses, it is considered safe and effective (FDA 2011). The FDA has limited the strength of APAP to 325 mg per tablet/capsule, in addition to assigning a ‘black box’ warning for severe hepatocellular injury to help protect consumers from APAP overdose (Babai et al. 2018; FDA 2014; Holt and Ju 2006). Despite these efforts, APAP overdose remains one of the primary causes of acute liver failure in the United States (Babai et al. 2018; Holt and Ju 2006; Larson et al. 2005). As approximately 50% of APAP overdose cases are unintentional, early detection biomarkers for DILI would allow clinicians to identify APAP overdose early and administer *N*-acetylcysteine (NAC) to prevent serious or any further progression of hepatocellular injury (Babai et al. 2018; FDA 2014; Holt and Ju 2006).

### The issue at hand—new biomarkers for DILI need to be identified

Consequently, due to the current preclinical and clinical limitations of traditional biomarkers for DILI, new and improved biomarkers for DILI are required. Not only will they need to be more specific and sensitive in diagnosing DILI, particularly early-stage DILI, but will also need to provide insight into the mechanism of hepatocellular injury. Ideally, these new biomarkers would be deployed in both preclinical and clinical settings, and would need to fill the current gaps left by traditional biomarkers for DILI.

Cytokeratin-18 (CK18) is one of a handful of potential biomarkers for DILI**.** CK18 is found in the intermediate filaments of the liver and has been identified as a potential biomarker for DILI (Tajima et al. 2019). CK18 and its relationship with hepatocellular injuries, such as DILI, has been extensively investigated in multiple clinical settings. However, in vivo rat models of hepatotoxicity investigating potential biomarkers (such as miRNAs and glutamate dehydrogenase) for DILI have not included CK18 in their investigative panels (Bailey et al. 2012, 2018). This is due to the lack of good, quantitative assays which has contributed to the lack of qualification for CK18. It is important to characterise CK18 and its relationship with hepatotoxicity in in vivo rat models, as these are heavily used for preclinical drug development. Detecting a signal in preclinical testing that is also monitorable in the clinic would help guide clinicians through a drug’s development safely.

Therefore, this review will highlight the properties of CK18 that may help to fill current gaps in knowledge left by traditional biomarkers for DILI, provide a brief overview of recent clinical research and discuss the current challenges and limitations surrounding the characterisation of CK18 in an in vivo rat model of hepatotoxicity.

### Cytokeratin-18 (CK18)

The cellular location of CK18, also referred to as KRT18, and the cleavage patterns of the protein make it a potential biomarker for DILI. CK18 is a type-I intermediate filament protein highly concentrated in hepatocytes and cholangiocytes (epithelial cells of the bile duct), comprising 5% of total liver protein (Tajima et al. 2019; Uhlén et al. 2015). The acidic protein contains a central helical rod domain flanked by a N-terminal head and C-terminal tail region and is co-expressed with type-II intermediate filament protein cytokeratin-8 (CK8) (Omary et al. 2006; Schutte et al. 2004). CK8/CK18 heterodimers are a resilient and adaptable scaffold for hepatocytes, with the ability to endure mechanical and nonmechanical stresses, such as those encountered during DILI (Coulombe and Omary 2002). The importance of CK18 in the liver has been highlighted in CK18 knockout mice, with the absence of CK18 in hepatocytes leading to the spontaneous development of liver lesions (closely reflecting the morphological spectrum of steatohepatitis-associated liver carcinogenesis), as well as liver tumours (Bettermann et al. 2016).

### CK18 may address the current gaps in knowledge and limitations left by traditional biomarkers for DILI

As well as providing a vital scaffold for epithelial cells of the liver, CK18 may help to address some of the current gaps in knowledge and limitations of traditional markers for DILI.

### CK18 can identify the mechanism of hepatocellular injury

**Fig. 1:**
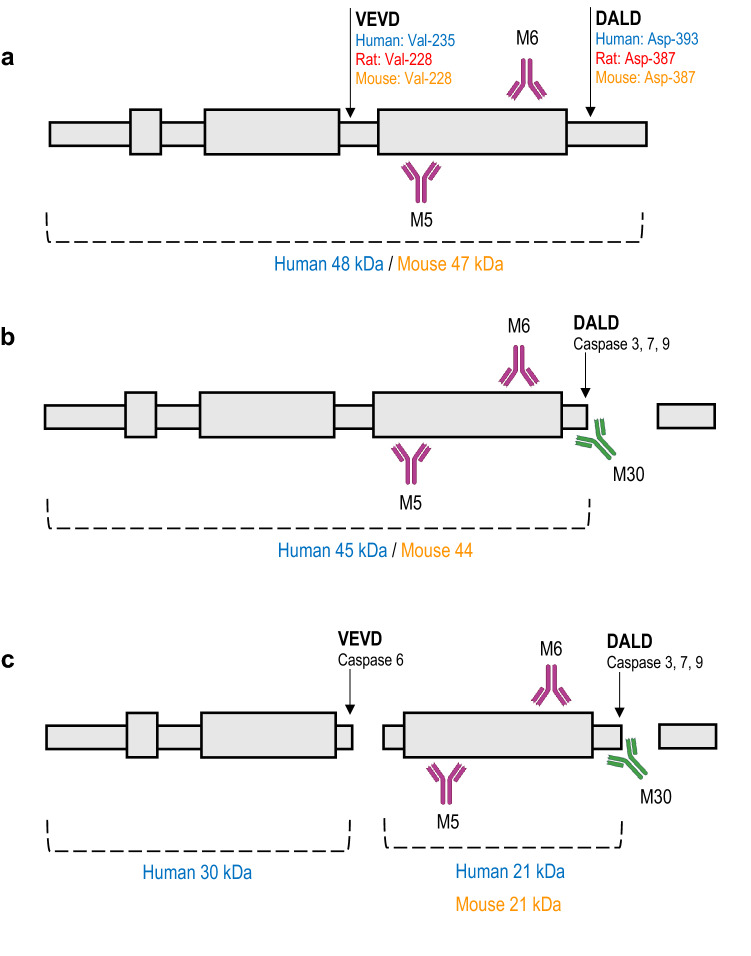
A schematic representation of full-length CK18 (**a**) and ccCK18 fragments (**b**, **c**). **a** Full-length CK18 contains two caspase consensus sites, VEVD and DALD. Full-length CK18 is recognised by the M5 and M6 antibody which is deployed in the M65 ELISA. The position of VEVD and DALD, as well as the molecular weight of the CK18 protein, is shown. **b** Following cleavage at the DALD site by caspases 3, 7 and 9, two ccCK18 fragments are generated. Cleavage at the DALD site is recognised by the M30 antibody deployed in the M30 ELISA. The M6 and M5 antibody recognition sites remain conserved. **c** Following cleavage at both DALD and VEVD sites, three ccCK18 fragments are generated. The M6, M5 and M30 antibody recognition sites remain conserved

Traditional diagnostic markers provide limited insight into the mechanism of hepatocellular injury whereas, the level of both full-length CK18 and caspase-cleaved CK18 (ccCK18) fragments in serum or plasma reflects the degree of necrotic hepatocellular injury and/or apoptosis (Church and Watkins 2017). During acute and chronic hepatocellular injury, necrotic cells passively release full-length CK18 into circulation due to the loss of cell membrane integrity (Caulín et al. 1997; Church and Watkins 2017; Schutte et al. 2004). In apoptosis, CK18 is targeted for proteolysis to facilitate the breakdown of the cytoskeleton and is released into circulation as ccCK18 stable fragments (Caulín et al. 1997; Church and Watkins 2017; Fadok and Henson 1998; Schutte et al. 2004). As demonstrated in Fig. 1, the CK18 protein contains two caspase consensus sites, DALD and VEVD. The DALD motif is located in the C-terminal tail region and is targeted by caspases 3, 7 and 9 immediately following early apoptotic events such as the loss of membrane potential, presence of DNA fragmentation and release of cytochrome *c* (Fig. 1b, c) (Caulín et al. 1997; Ku et al. 1997; Leers et al. 1999; Schutte et al. 2004). The VEVD motif is located in the central helical rod domain and is solely targeted by caspase 6, with cleavage of the VEVD motif responsible for the final collapse of the CK18 cytoskeleton (Fig. 1c) (Ku et al. 1997; Schutte et al. 2004).

In clinical settings, both full-length CK18 and ccCK18 fragment levels can be readily quantified by immunoassays. The locations of caspase cleavage and the molecular size of generated ccCK18 fragments have been identified by western blotting and liquid chromatography with tandem mass spectrometry (LC MS/MS) in both clinical and in vivo mouse models of DILI (Micha et al. 2008; Schutte et al. 2004).

### The detection of necrotic hepatocellular injury with full-length CK18 can diagnose early-stage DILI

The most significant advantage of utilising CK18, in particular full-length CK18, as a biomarker for DILI is that it can diagnose early-stage DILI (Church and Watkins 2017). The level of some traditional biomarkers, such as ALT and AST, are often elevated without the presence of any hepatocellular injury. Therefore, significant fold increases in ALT and/or AST levels (> 3–5 times the ULN) are considered adverse and indicative of potential hepatocellular injury. However, these significant elevations generally occur during the later stages of hepatocellular injury, when serum levels of these enzymes rise as liver function becomes increasingly impaired (Church and Watkins 2017). As serum levels of full-length CK18 are indicative of necrosis, detecting full-length CK18 in serum can indicate early necrotic hepatocellular injury (Church and Watkins 2017). However, given the ubiquitous expression of CK18, the abundance of full length CK18 needs to be standardised to either traditional diagnostic makers or potentially new biomarkers.

### Identifying necrosis and apoptosis provides insight into the involvement of inflammation during hepatocellular injury

Identifying the mechanism of hepatocellular injury is key to assess the severity of DILI and enables the early administration and implementation of treatments and interventions which may improve prognosis. Necrosis is a process predominately driven by the innate immune response mediated by Toll-like receptors and subsequently, pro- and anti-inflammatory cytokines such as tumour necrosis alpha (TNF-α) and interlukin-1 alpha (IL-1α) (Takeda and Akira 2015; Yang et al. 2015; Yilmaz 2009). On the other hand, apoptosis is programmed cell death that predominately occurs through either activation of the TNF superfamily (extrinsic pathway) or the presence of free radicals (intrinsic pathway) (Caulín et al. 1997; Lorente 2018; Yilmaz 2009). In patients experiencing non-severe and severe idiosyncratic DILI, the serum level of pro- and anti-inflammatory cytokines in combination with serum levels of full-length CK18 and ccCK18 fragments were shown to be able to determine which mechanism of hepatocellular injury, either necrosis or apoptosis, predominated in each patient group (Xie et al. 2019). In patients experiencing non-severe idiosyncratic DILI, serum levels of TNF-α, IL-1α, macrophage inflammatory protein 1-beta (MIP-1β) and interferon gamma-induced protein 10 (IP-10) were significantly increased whilst serum levels of ccCK18 fragments were significantly decreased in comparison to patients experiencing severe idiosyncratic DILI (*P* < 0.05) (Table 1) (Xie et al. 2019). With these findings, it was concluded that necrotic hepatocellular injury was more predominate in patients experiencing non-severe idiosyncratic DILI compared to patients experiencing severe idiosyncratic DILI (Xie et al. 2019).

**Table 1:** A summary of recent clinical studies that utilised full-length CK18 and ccCK18 fragments to detect hepatocellular injury^a^

Reference	Hepatocellular injury	CK18 quantification	Findings
(Church et al. 2019)	DILI (Various causative agents) Serum samples were collected from the DILIN, PSTC and SAFE-T patient cohorts	Human M30 and M65 ELISA (Peviva, Sweden)	Elevated serum full length CK18 and ccCK18 were significant predictors for death/liver transplantation (ROC AUC 0.832, 95% CI 0.737–0.927 for full length CK18 and ROC AUC 0.778, 95% CI 0.676–0.881 for ccCK18) Serum full length CK18 and ccCK18 were more sensitive and predictive for death/liver transplantation than AST (ROC AUC 0.700, 95% CI 0.587–0.814), ALT (ROC AUC 0.606, 95% CI 0.433–0.780) and ALP (ROC AUC 0.597, 95% CI 0.433–0.760) An AI was calculated for 162 patients utilising the ratio of serum full length CK18:ccCK18. AI was determined to be a significant predictor for death/liver transplantation (ROC AUC 0.761, 95% CI 0.627–0.895) Incorporating CK18 (in conjunction MCSFR) into MELD scoring increased the specificity of MELD scoring from 0.738 to 0.889. Sensitivity remained the same at 0.933 Biomarkers such as CK18 and ccCK18 were most altered in APAP-induced hepatoxicity DILIN patients with Augmentin-induced hepatoxicity ↑ serum full length CK18 and ccCK18 compared to SAFE-T patients (*P* = 0.028)
(Xie et al. 2019)	Idiosyncratic DILI (Various causative agents)	Human M30 and M65 ELISA (Peviva, Sweden)	Serum full length CK18 and ccCK18 ↑ in non-severe DILI and severe DILI vs control (*P* < 0.01) Serum ccCK18 ↑ in severe DILI vs non-severe DILI (*P* < 0.05) Serum full length CK18 correlated with serum ALT (*R*^2^ = 0.632, *P* < 0.001) and AST (*R*^2^ = 0.754, *P* < 0.001) levels Serum ccCK18 correlated with ALT (*R*^2^ = 0.554, *P* < 0.001) and AST (*R*^2^ = 0.657, *P* < 0.001) levels No significant differences in serum full length CK18:ccCK18 ratio between control, non-severe DILI and severe DILI (*P* > 0.05)
(Vatsalya et al. 2019)	AAH, AUD and NASH	Human M30 and M65 ELISA (Peviva, Sweden)	Serum full length CK18:ccCK18 ratio ↑ in severe AAH vs AUD (*P* < 0.05) and ↑ in severe AAH vs NASH (*P* < 0.05) Serum full length CK18:ccCK18 ratio ↑ in moderate AAH vs NASH (*P* < 0.05) Serum full length CK18 and ccCK18 were not correlated with ALT or AST levels (*P* > 0.05) Serum Full length CK18:ALT ratio ↑ in AHH vs NASH (*P* < 0.001) Serum ccCK18:ALT ratio ↑ in AHH vs NASH (*P* < 0.001)
(Godin et al. 2015)	HCC and cirrhosis (Various causes)	Human M30 and M65 ELISA (Enzo Life Sciences, France)	Serum full length CK18 ↑ in HCC vs cirrhosis (*P* < 0.05) Serum ccCK18 ↑ in HCC vs cirrhosis (*P* < 0.05) No significant differences in serum full length CK18:ccCK18 ratio in HCC vs cirrhosis (*P* > 0.05)
(Godin et al. 2015) In vitro*,* *Hu7cells*	DILI (Erastin, Doxorubicin and Sorafenib)	Human M30 and M65 ELISA (Enzo Life Sciences, France)	Cells treated with Doxorubicin ↑ full length CK18 vs control (*P* < 0.05) Cells treated with Erastin, Doxorubicin and Sorafenib ↑ ccCK18 vs control (*P* < 0.05) Full length CK18:ccCK18 ratio ↑ with Doxorubicin vs control (*P* < 0.05)
(Yagmur et al. 2007)	CLD (Various causes)	Human M30 (Peviva, Sweden)	Serum full length CK18 ↑ vs control (*P* < 0.001) Serum full length CK18 more prominent in the early stage of CLD Serum full length CK18 correlated with ALT (*R*^2^ = 0.602, *P* < 0.001), AST (*R*^2^ = 0.644, *P* < 0.001) and ALP (*R*^2^ = 0.397, *P* < 0.001) Serum full length CK18 superior to ALT, AST and ALP in distinguishing between no or mild hepatic injury vs severe hepatic injury (*P* < 0.01)

^a^*DILIN* Drug-induced Liver Injury Network, samples were collected from patients within 6 months of DILI onset from multiple centres within the United States, *PSTC*: Predictive Safety Testing Consortium, *SAFE-T* Safer and Faster Evidence-based Translation, *ELISA* enzyme-linked immunosorbent assay, *ROC AUC* receiver operator characteristic, area under the curve, *AI*: apoptotic index of injury, *MCSFR* macrophage colony-stimulating factor receptor, *MELD scoring* model for end-stage liver disease calculated as 9.57 × Log_e_(creatinine) + 3.78 × Log_e_ (total bilirubin) + 11.2 × Log_e_ (international normalised ratio) + 6.43; ↑:Significant increase, *NASH* Non-alcoholic steatohepatitis; vs: compared to, *AAH* acute alcoholic hepatitis; *AUD* alcohol use disorder; *HCC* hepatocellular carcinoma, *CLD* chronic liver disease

### How does CK18 and ccCK18 compare to other novel biomarkers for DILI?

CK18 and ccCK18 are not the only novel biomarkers to diagnose DILI, with glutamate dehydrogenase (GLDH), microRNA-122 (miRNA-122), macrophage colony stimulating factor receptor (MCSFR) and osteopontin (OPN) also current candidates (Church et al. 2019). We have favoured both CK18 and ccCK18 over the before-mentioned novel biomarkers as they are more sensitive and specific in diagnosing early-stage DILI, can identify the mechanism of hepatocellular injury and have demonstrated a strong and consistent relationship with hepatocellular injury in clinic. In comparison, liver-specific miRNA-122 has demonstrated large inter- and intra-patient variability, particularly amongst healthy patient cohorts, whilst GLDH has been recommended for use in certain clinical cohorts (Church et al. 2019; Flanigan et al. 2014). Similarly, both MCSFR and OPN are primarily inflammatory markers therefore, do not provide insight into the mechanism of hepatocellular injury and can also be indicative of wide-spread inflammation (Church et al. 2019).

### Clinical research has identified a strong relationship between serum levels of full-length CK18 and ccCK18 fragments with hepatocellular injury

A summary of clinical studies that utilised the serum levels of full-length CK18 and ccCK18 fragments to diagnose DILI are outlined in Table 1. Additionally, clinical studies investigating hepatocellular injuries non-alcoholic steatohepatitis (NASH), acute alcohol hepatitis (AAH) and hepatocellular carcinoma (HCC) were also included. As evident from Table 1, in each study serum levels of full-length CK18 and ccCK18 fragments were significantly increased in patients with hepatocellular injury compared to healthy controls (*P* < 0.05). In a handful of these studies, comparisons between the serum level of full-length CK18, ccCK18 fragments and traditional diagnostic markers ALT and AST were made (Table 1) (Gonzalez-Quintela et al. 2006; Vatsalya et al. 2019; Xie et al. 2019; Yagmur et al. 2007). The serum level of full-length CK18 and ccCK18 fragments were correlated to serum levels of ALT and AST in patients with chronic liver disease or idiosyncratic DILI (*P* < 0.05) (Table 1) (Gonzalez-Quintela et al. 2006; Xie et al. 2019; Yagmur et al. 2007). However, in patients with AAH, AUD or NASH, there was no correlation between serum level of full-length CK18 and ccCK18 fragments with ALT or AST (*P* < 0.01) (Table 1) (Vatsalya et al. 2019). These results suggest the utility of both full-length CK18 and ccCK18 fragments as biomarkers for hepatocellular injury is dependent on the type of hepatocellular injury.

The ratio of full-length CK18:ccCK18 was also determined to identify the proportion of hepatocellular injury attributable to apoptosis compared to necrosis (Church et al. 2019; Godin et al. 2015; Vatsalya et al. 2019; Xie et al. 2019). In Table 1, it was identified that as the severity of hepatocellular injury increased, the ratio of CK18:ccCK18 also increased, indicating apoptosis became more prominent as the severity of injury increased (*P* < 0.05) (Church et al. 2019; Godin et al. 2015; Vatsalya et al. 2019; Xie et al. 2019).

Furthermore, predictive risk modelling identified serum levels of both full-length CK18 and ccCK18 fragments (receiver operator characteristic area under the curve (ROC AUC) = 0.83 and 0.78, respectively) were more sensitive and specific in predicting the prognosis of death and liver transplantation compared to serum levels of AST, ALT and ALP (ROC AUC = 0.70, 0.61 and 0.60, respectively) (Church et al. 2019). Serum full length CK18 was also found to be superior in distinguishing between mild and severe hepatocellular injury in chronic liver disease compared to serum levels of ALT, AST and ALP (*P* < 0.01) (Yagmur et al. 2007).

### There are various challenges and limitations surrounding the characterisation of CK18 in preclinical models of DILI

Clinical research has demonstrated a strong relationship between serum levels of full-length CK18 and ccCK18 fragments with DILI. However, to improve our interpretation of both full-length CK18 and ccCK18 fragments as biomarkers for DILI and for adding value in both clinical and preclinical settings, CK18 must be better characterised in preclinical models of DILI. Due to an array of challenges, only a handful of preclinical in vivo rat studies have investigated CK18 as a potential biomarker for hepatotoxicity. A summary of these in vivo rat studies and their limitations is outlined in Table 2. Of the current challenges and limitations surrounding the characterisation of CK18 as a biomarker of DILI in rat, the most significant is the lack of a species-specific quantitative CK18 assay.

**Table 2:** A summary of in vivo rat models that utilised full-length CK18 and ccCK18 fragments to detect hepatocellular injury^2^

Study details	CK18 quantification	Findings	Limitations of the study
**(**Dai et al. 2020**)**			
Sprague Dawley rats with NASH (high-fat diet)	Human M30 ELISA (Shanghai Biotechnology, China)	Serum ccCK18 ↑ in NASH vs control (*P* = 0.035) Serum ccCK18 correlated with liver pathological scores (*R*^2^ = 0.631, *P* = 0.008) No correlation between serum ALT levels and liver pathological scores (*P* = 0.055)	Human M30 ELISA has 100-fold lower efficiency in rats Intra-assay variability of the M30 ELISA
**(**Maliver et al. 2017**)**			
Wistar rats with DILI (Clofibrate)	Human M30 ELISA (MyBioSource, USA)	Serum ccCK18 ↑ at 400 and 750 mg/kg clofibrate dose vs control (*P* < 0.05 and *P* < 0.01) Serum ALT levels ↑ at 200, 400 and 750 mg/kg clofibrate dose vs control (*P* < 0.01, *P* < 0.001 and *P* < 0.001, respectively) Correlation between liver biomarkers and hepatocyte hypertrophy was not conducted	Human M30 ELISA has 100-fold lower efficiency in rats Intra-assay variability of the M30 ELISA Clofibrate is not a commonly used in vivo model of DILI
**(**Kakehashi et al. 2009**)**			
Fisher 344 rats with DILI [*N*-Nitrosodimethylamine (DEN) and Phenobarbital (PB)]	IHC and RT Q-PCR	IHC demonstrated full length CK18 ↑ DILI (DEN + PB) vs control (*P* < 0.001) Full length CK18 mRNA ↑ in DILI (DEN + PB) vs control (*P* < 0.05) ccCK18 was not investigated	RT Q-PCR is indicative of gene expression. No protein analysis IHC is semi-quantitative

^b^*NASH* non-alcoholic steatohepatitis, *ELISA* enzyme-linked immunosorbent assay,↑Significant increase, vs: compared to, *IHC* immunohistochemistry, *RT Q-PCR* real-time quantitative polymerase chain reaction

### At present, the serum and plasma level of full-length CK18 and ccCK18 fragments cannot be quantified in an in vivo rat model of DILI

**Fig. 2:**
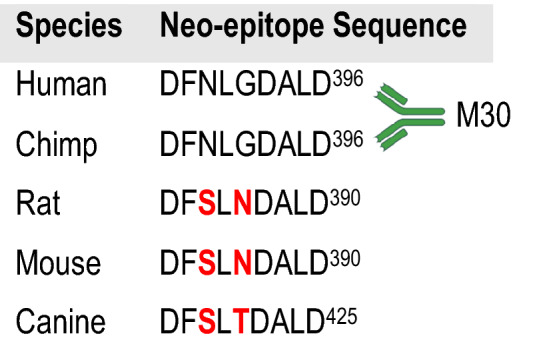
The neo-epitope produced in human, chimp, rat, mouse and canine CK18 following cleavage at the DALD cleavage site by caspases 3, 7 and 9. The M30 antibody recognises the human neo-epitope however, it has a 100-fold lower efficiency for the rat and mouse neo-epitope

Due to the current lack of rat-specific quantitative assays, semi-quantitative and qualitative methods such as western blotting and LC MS/MS need to be used to identify the presence of full-length CK18 and ccCK18 fragments in in vivo rat models of DILI. In clinical research, the serum level of full-length CK18 and ccCK18 fragments is quantified using the M65 and/or M30 ELISA (Fig. 1 and Table 1). The M65 ELISA, utilizing the M5 and M6 CK18 monoclonal antibodies, detects binding to epitopes of the CK18 protein that are present in both full-length CK18 and ccCK18 fragments (Fig. 2B, C) (Kramer et al. 2004; Olofsson et al. 2009). Hence, the M65 ELISA can quantify the total level of CK18 (U/L), measuring total cell death by necrosis (full-length CK18) and apoptosis (ccCK18 fragments) (Kramer et al. 2004; Olofsson et al. 2009). Currently, M65 is only specific for human CK18 and although a number of rat-specific M65 ELISAs have recently been released for commercial use, their performance is yet to be reported in the literature.

The M30 ELISA is also used in clinical research and this quantifies the serum level of ccCK18 fragments (Olofsson et al. 2009; Pimentel et al. 2016). The M30 antibody recognises the neo-epitope generated following cleavage at the DALD site in human CK18 at amino acid position 393 (Fig. 2B, C) (Pimentel et al. 2016). The M30 ELISA solely quantifies apoptosis and by examining both M65 and M30 ELISAs in parallel, the degree of necrotic hepatocellular injury and apoptosis can be determined. The region recognised by the M30 ELISA is 87% conserved between human and mouse CK18 (Olofsson et al. 2009). However, research has shown the peptide used to compete for CK18 in the M30 ELISA has a 100-fold lower efficiency for both mouse and rat CK18 due to changes in the amino acid sequence as demonstrated in Fig. 2 (Olofsson et al. 2009).

Additionally, poor intra-assay variability is a common problem identified between M30 ELISAs. Pimentel et al. (2016) measured serum levels of ccCK18 fragments in a cohort of NASH patients using two commercially available M30 ELISA kits (Pimentel et al. 2016). It was determined that there was no significant correlation between serum levels of ccCK18 fragments between the two M30 ELISA kits (*P* = 0.86, *r* = 0.01) (Pimentel et al. 2016). Furthermore, binary logistic regression only identified the serum level of ccCK18 fragments quantified by one of the M30 ELISAs to be a significant predictor for NASH (Pimentel et al. 2016).

Until a rat-specific M65 ELISA is optimised, semi-quantitative and qualitative methods will need to be utilised to detect full-length CK18 and ccCK18 fragments in an in vivo rat model of DILI. The significant disadvantage of using semi-quantitative and qualitative methods is they cannot be used to compare values across studies or to compare the full length CK18 to ccCK18 in a meaningful way. Therefore, to implement both full-length CK18 and ccCK18 fragments as routine biomarkers for DILI in preclinical settings, it is imperative a rat-specific M65 ELISA is optimised and a rat-specific M30 ELISA is developed. In addition, proteomic-based analysis such as LC MS/MS and SWATH MS (sequential window acquisition of all theoretical fragment ion spectra mass spectrometry) may also be useful to quantitate the levels of full length CK18 and ccCK18 fragments. However, these forms of analyses will only be beneficial once rat CK18 protein has been characterised and added to the respective protein libraries. Once full length CK18 and ccCK18 fragments are able to be quantified, the next step is selecting the appropriate in vivo rat model of DILI. However, this in itself has its own challenges.

### The commonly used acetaminophen (APAP) in vivo model of DILI has notable disadvantages in rats

The two most common model hepatotoxins used in in vivo research are APAP and carbon tetrachloride (CCl_4_) nevertheless, both models have specific limitations in rats, the species commonly used in preclinical drug safety testing (McGill and Jaeschke 2019). APAP is the preferred in vivo model of DILI due to its clinical relevance and the fact that the mechanism of hepatocellular injury induced by APAP is well understood (McGill and Jaeschke 2019; McGill et al. 2012b). APAP overdose is a leading cause of acute liver failure in several Western Countries, including the United Kingdom and United States, with hepatocellular injury mediated by NAPQI (Bernal 2003; McGill and Jaeschke 2019; Ostapowicz et al. 2002). The conversion of APAP to NAPQI is catalysed by cytochrome P450 enzymes (McGill et al. 2012a; Xie et al. 2015). NAPQI subsequently binds to sulfhydryl groups on glutathione (GSH) and proteins, depleting glutathione and increasing cell susceptibility to oxidative stress (McGill et al. 2012a; Xie et al. 2015). Additionally, NAPQI binds to mitochondrial proteins, inhibiting mitochondrial respiration and leading to the development of mitochondrial oxidative stress (Cover et al. 2005; Meyers et al. 1988).

The most significant concern of using APAP in in vivo rat models of DILI is that rats are more resistant to APAP-induced hepatocellular injury (McGill et al. 2012b). When administered comparable doses of APAP, the degree of hepatocellular damage and hepatotoxicity in rats is limited and, in some instances, non-existent, compared to the degree of hepatocellular damage observed in humans (McGill et al. 2012b). The mechanism of APAP toxicity in humans is more similar to mice, with doses of ≥ 150 mg/kg inducing DILI in both species (Boxill et al. 1958; Eder 1964; Jaeschke et al. 2014; McGill et al. 2012b). However, some reports in Sprague–Dawley and Fisher rats treated with APAP at 1–2 g/kg suggest there was no evidence of oxidative stress with no significant differences in GSH/oxidised glutathione (GSSG) percentage following APAP treatment (*P* > 0.05) (McGill et al. 2012b). Mitochondrial APAP-protein adduct levels were also not significantly different compared to control treated rats (*P* < 0.05) (McGill et al. 2012b). Furthermore, no histological hepatocellular injury was identified in these rats following APAP treatment (McGill et al. 2012b). It is unclear why rats are resistant to APAP-induced hepatocellular injury but, it is crucial that a significant degree of hepatocellular injury is present to adequately investigate the relationship between serum levels of full-length CK18 and ccCK18 fragments with DILI.

CCl_4_ is also a commonly used in vivo model for DILI research. CCl_4_ is a chlorinated hydrocarbon with high doses of CCl_4_ ≥ 1 mL/kg inducing hepatocellular injury that resembles intrinsic DILI (McGill and Jaeschke 2019). Unlike APAP, the mechanism of CCl_4_ hepatocellular injury is not well understood but, is believed to be dependent on the reactive metabolite trichloromethyl radical (CCl_3_) (McGill and Jaeschke 2019; Weber et al. 2003). CCl_4_ is converted by cytochrome P450 to CCl_3_, which subsequently binds to proteins, DNA and lipids, leading to mitochondrial and oxidative stress (McGill and Jaeschke 2019). Necrosis induced by CCl_4_ is limited to areas of high cytochrome P450 concentration and expression, such as the centrilobular area of the liver (Weber et al. 2003). This specific pattern of hepatocellular injury is not consistent with other forms of DILI but, most importantly, as CCl_4_ is not a pharmaceutical drug, it may be difficult to translate the results of a CCl_4_ in vivo rat model of DILI (Slater 1966; Weber et al. 2003).

Another potential but less common in vivo rat model for DILI is methotrexate (MTX). MTX is an antimetabolite drug used for the treatment and maintenance of inflammatory diseases (low-dose MTX, 7.5–25 mg daily) and forms part of many chemotherapy regimens (high-dose MTX, 200–800 mg bolus doses) (Sotoudehmanesh et al. 2010; Whirl-Carrillo et al. 2012). Hepatocellular injury is the most common adverse effect of high-dose MTX, with hepatocellular damage localised in liver sinusoidal endothelial cells (Sotoudehmanesh et al. 2010). High-dose MTX in vivo significantly elevates ALT and AST levels, increases expression of fibrin and, leads to severe steatosis, sinusoidal dilation, as well as, moderate inflammation and necrosis (Ewees et al. 2019). Given the clinical nature of MTX and strong, reproducible evidence MTX induces severe hepatocellular injury in vivo, MTX may be favoured over traditional APAP and CCl_4_ in vivo rat models of DILI.

In addition to selecting the appropriate in vivo rat model of DILI, one must also understand how liver regeneration in the selected model may affect the release of full-length CK18 and ccCK18 fragments into circulation.

### Liver regeneration in the selected in vivo model of DILI may affect the kinetics of full-length CK18 and ccCK18 fragments

Liver regeneration in the chosen in vivo rat model of DILI can also be considered. The liver has the unusual capacity to repair and regenerate following hepatocellular injury and partial hepatectomy (Clemens et al. 2019). Upon DILI, an intricate signalling process mediated by cytokines, chemokines and growth factors is triggered to stimulate healthy hepatocytes surrounding areas of necrosis to enter the cell cycle and undergo division (Apte et al. 2009; Clemens et al. 2019; Leevy et al. 1959). Following APAP-induced hepatocellular injury, TNF-α and interleukin-6 (IL-6), as well as ß-catenin, endothelial growth factor receptor (EGFR) and vascular endothelial growth factor (VEGF), play a vital role in liver regeneration (Bhushan and Apte 2019; Donahower et al. 2006). Similarly, TNF-α, IL-6 and hepatocellular growth factor (HGF) are also involved in liver regeneration following CCl_4_-induced hepatocellular injury (Burr et al. 1998; Clemens et al. 2019; Scheving et al. 2015). Liver regeneration can prevent the progression of DILI therefore, it is critical a dose of APAP or CCl_4_ is administered that inhibits liver regeneration and leads to the rapid progression of hepatocellular injury (Mehendale 1991, 2005).

### The tissue specificity of CK18 needs to be established in both preclinical and clinical models

CK18 is highly concentrated in hepatocytes and cholangiocytes however, as the protein plays a vital role in maintaining the cytoskeleton of epithelial cells, it can also be found in epithelial cells lining other organs (Church et al. 2019; Church and Watkins 2017). Two-dimensional gel electrophoresis observed CK18 in substantial amounts in simple cuboidal epithelial cells lining the pancreatic ducts and kidney tubules, as well as simple columnar epithelial cells lining the mucosa of the small intestine and colon (Ku et al. 1999; Moll et al. 1982). The pancreas, kidneys, small intestine and colon are all known to be targets of drug-induced injury, particularly following therapy with angiotensin-converting enzyme inhibitors (pancreas), non-steroidal anti-inflammatories (kidneys) and cytotoxic drugs (small intestine and colon). As CK18 has been identified in a handful of susceptible organs, in some instances of multi-organ drug-induced toxicity, it may be difficult to determine if serum levels of full-length CK18 and ccCK18 fragments are only reflective of DILI or, reflect an array of drug-induced injuries (Church and Watkins 2017; Tajima et al. 2019).

CK18 is also expressed by a variety of adenocarcinomas such as those of the lung, pancreas, prostate, colon and rectum (Kramer et al. 2004). During cytotoxic drug therapy, CK18 is released from tumour cells and plasma levels of both full-length CK18 and ccCK18 fragments are commonly utilised to evaluate clinical progression and tumour cell death (Kramer et al. 2004). This may present challenges when one or multiple cytotoxic drugs are used in therapy that also induce DILI, such as irinotecan or oxaliplatin (Robinson et al. 2012).

As such, it is important for both preclinical and clinical models to determine the level of CK18 present in organs such as the kidney and small intestine and if full-length CK18 and/or ccCK18 fragments are released during drug-induced injury from these organs. This can be addressed in preclinical models of drug-induced kidney, pancreatic and gastrointestinal injury. It may be necessary to define a predetermined ratio or percentage of full-length CK18 and ccCK18 fragments that can be attributed to hepatocellular injury (Church and Watkins 2017). Nevertheless, CK18 may still address current gaps in knowledge and limitations, in particular, forming part of a biomarker panel that can inform on necrotic and apoptotic hepatocellular injury and tissue specificity.

## Conclusions

DILI is a frequent and serious adverse reaction that can occur during preclinical and clinical drug therapy and for which new biomarkers are required. CK18 is a potential biomarker for DILI and has some desirable properties that may help in detection of early-stage DILI and in the identification of the mechanism of hepatocellular injury. CK18 has been applied as a biomarker for DILI in clinical research, demonstrating both full-length and cleaved versions of the protein are accurate and sensitive in diagnosing DILI. However, for CK18 to be applied as a biomarker for DILI preclinically, it needs to be characterised in an in vivo rat model of DILI, due to being the routine species used in preclinical drug safety assessment. The challenges of characterising CK18 in an in vivo rat model of DILI are well-documented, but, once the appropriate in vivo model of DILI has been identified, it will be possible to undertake validation work on recently released rat-specific M65 ELISAs, and determine the specificity of CK18 to hepatocellular injury. This review has provided evidence to support the characterisation of CK18 in an in vivo rat model of DILI, and to investigate the potential translation of CK18 as a routinely used biomarker for DILI in clinical settings.

## Data Availability

Not applicable.
